# Assessing Opportunities and Barriers to Improving the Secondary Use of Health Care Data at the National Level: Multicase Study in the Kingdom of Saudi Arabia and Estonia

**DOI:** 10.2196/53369

**Published:** 2024-08-08

**Authors:** Janek Metsallik, Dirk Draheim, Zlatan Sabic, Thomas Novak, Peeter Ross

**Affiliations:** 1 E-Medicine Centre, Department of Health Technologies School of Information Technologies Tallinn University of Technology Tallinn Estonia; 2 Information Systems Group School of Information Technologies Tallinn University of Technology Tallinn Estonia; 3 Health, Nutrition and Population Global The World Bank Group Washington, DC United States; 4 Office of the National Coordinator for Health Information Technology Department of Health and Human Services Washington, DC United States; 5 Research Department, East Tallinn Central Hospital Tallinn Estonia

**Keywords:** health data governance, secondary use, health information sharing maturity, large-scale interoperability, health data stewardship, health data custodianship, health information purpose, health data policy

## Abstract

**Background:**

Digitization shall improve the secondary use of health care data. The Government of the Kingdom of Saudi Arabia ordered a project to compile the National Master Plan for Health Data Analytics, while the Government of Estonia ordered a project to compile the Person-Centered Integrated Hospital Master Plan.

**Objective:**

This study aims to map these 2 distinct projects’ problems, approaches, and outcomes to find the matching elements for reuse in similar cases.

**Methods:**

We assessed both health care systems’ abilities for secondary use of health data by exploratory case studies with purposive sampling and data collection via semistructured interviews and documentation review. The collected content was analyzed qualitatively and coded according to a predefined framework. The analytical framework consisted of data purpose, flow, and sharing. The Estonian project used the Health Information Sharing Maturity Model from the Mitre Corporation as an additional analytical framework. The data collection and analysis in the Kingdom of Saudi Arabia took place in 2019 and covered health care facilities, public health institutions, and health care policy. The project in Estonia collected its inputs in 2020 and covered health care facilities, patient engagement, public health institutions, health care financing, health care policy, and health technology innovations.

**Results:**

In both cases, the assessments resulted in a set of recommendations focusing on the governance of health care data. In the Kingdom of Saudi Arabia, the health care system consists of multiple isolated sectors, and there is a need for an overarching body coordinating data sets, indicators, and reports at the national level. The National Master Plan of Health Data Analytics proposed a set of organizational agreements for proper stewardship. Despite Estonia’s national Digital Health Platform, the requirements remain uncoordinated between various data consumers. We recommended reconfiguring the stewardship of the national health data to include multipurpose data use into the scope of interoperability standardization.

**Conclusions:**

Proper data governance is the key to improving the secondary use of health data at the national level. The data flows from data providers to data consumers shall be coordinated by overarching stewardship structures and supported by interoperable data custodians.

## Introduction

### Background

Governments seek guidance and strategic directions for deploying effective, efficient, and reliable mechanisms for the secondary use of data collected in health care provision. While the primary use of digital data in health care institutions has developed well during the last decades, health care systems look to improve their practice for secondary use. The secondary use of data controls the burden of data capture by enabling the reuse of already collected data for alternative purposes. Among others, the categories of secondary data use include improving the patient experience, health care facility management, service planning and benchmarking, policy development, public health, health care financing, research, and business support [1]. The categories above exploit data traditionally collected in separate data streams and silos. For instance, public health registries or health insurance claims are managed in most countries by dedicated organizations within their databases using specific data collection processes. The siloed approach has led to the duplication of data collection and the waste of health care resources. A report by the Open Data Institute from 2021 concludes that initiatives for health data ecosystems for data reuse are still fragmented in Europe [2].

Digital data and digitalized processes allow for a change in these practices, making data capture universal and allowing digital health care data sharing for different purposes.

From 2019 to 2021, we conducted projects in the Kingdom of Saudi Arabia and Estonia, assessing health and health care data analytics and developing context-specific recommendations. The governments of both countries were looking to advance their decision-making capabilities due to the digitalization of the flow of health data.

### The Project in the Kingdom of Saudi Arabia

The Saudi Health Council (SHC), in cooperation with the World Bank, developed the National Master Plan for Health Data Analytics to guide and provide strategic direction for the deployment of effective, efficient, and reliable mechanisms to share data from the health sector for policy and decision-making [3].

The government sought to boost the regulatory, institutional, and technical infrastructure, allowing for efficient data collection from health care systems to process and provide appropriate data analytics and business intelligence for policy and decision makers. The project assessed the existing situation and conceptualized the harmonized national health data analytics operational model and the logical architecture, including core elements such as the Health Data Analytics Framework, actors and their roles, and critical processes.

The initial driver for the development was perceived inefficiency and observable delays in producing analytical data products about the country’s health care system. Indirectly, the existing data flow was limiting the ability to produce accurate and timely information for decision-making on many levels of the health care system. The project focused on the requirements of the significant national-level decision makers, including the SHC and management of the health care sectors, namely, Ministry of Health (MoH) Medical Services, National Guard Medical Services, Ministry of Interior Medical Services, and King Faisal Specialist Hospital & Research Center.

### The Project in Estonia

The analysis of health and health care data management was part of the Structural Reform Support Service mission of the European Commission, to provide support for the preparation and implementation of growth-enhancing administrative and structural reforms by mobilizing European Union funds and technical expertise. Estonia requested support from the European Commission under Regulation (EU) 2017/825 on the establishment of the Structural Reform Support Program (“SRSP Regulation”) to prepare the Person-Centered Integrated Hospital Master Plan [4].

The master plan targeted to (1) provide a map of the current hospital system, its ability to supply health care in different specialties, distribution of its physical and human resources, its financial flows, and its mechanisms of governance and information sharing; (2) provide evidence-based estimates of the population needs and the supply of health workforce and health care services and infrastructures; and (3) propose a hospital master plan of sound reforms in the hospital sector in the midterm.

The planning included an assessment of the data sharing mechanisms and governance. Current and future organizations delivering the data for decision-making throughout the hospital network were analyzed. The scope of the analysis included data for the national-level health care management and policy (Ministry of Social Affairs [MoSA]), public health (National Institute for Health Development [NIHD]), health care financing (Estonian Health Insurance Fund [EHIF]), and health care service management and clinical decision-making (hospitals and family health centers).

### The Tension Between Demand and Supply of Information

In both cases, the digitalization of the health and medical data flows should improve the quality of the decisions. Data in health care are often produced and consumed by different stakeholders. They need to cross the boundaries of specialties, institutions, regions, and sectors to deliver informational value to data consumers so that they can make decisions. From the point of view of decision makers, the place of capture has a surplus, and the place of decision-making has a shortage of information. The tension of disbalance generates the need for data flow: data consumers need data from data producers to extract information for decision-making.

The stakeholders in health care that need data for decision-making, such as governmental organizations, payers, policy makers, and others, feel the tension and try to resolve it. They request data providers to collect and deliver data for each type of consumption. As the providers cannot always align the requirements, they often capture the same information multiple times. The uncoordinated design of data flows has manifested in duplication, gaps, and delays.

The projects analyzed health care data supply and demand for data for different health care decisions. The analysis aimed to provide a better basis for planning data management organization and infrastructure.

**Figure 1:**
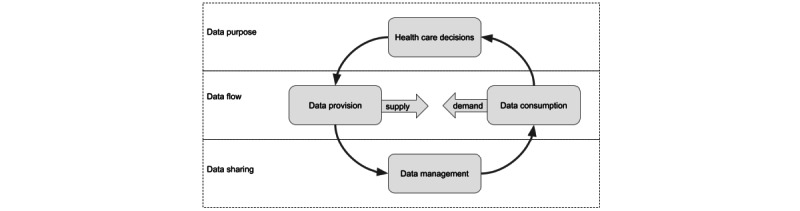
Health care decisions are the purpose of data consumption. Health care decisions also provide new data. Often, the source location of the provided data does not match the desired location of the consumed data. This disbalance between supply and demand creates tension that forces data to flow. Data management resolves the tension by sharing data to required locations.

In both cases, the client saw issues producing proper analytical data products. In the Kingdom of Saudi Arabia case, the focus was on the reports on public health and health care system indicators. The Estonian terms underlined person centricity and efficacy, which introduced the requirement to study data sharing for clinical decision-making, patient engagement, hospital management, health care system planning, health care policy, and health care funding. To assess the situation and plan for better data sharing, we found it essential to map the providers and consumers and evaluate the usability and use of data for decision-making. The assessment of health care data systems focused on the data purpose, flow, and sharing (Figure 1).

### The Aim of the Study

This study aimed to report problems and outcomes from the 2 distinct projects that assess the potential of secondary use of health care data and support of governmental decisions and to map the common thread of thought to apply in similar circumstances. We looked for the matching elements of the problems and the results of the 2 assessments.

## Methods

### Overview

The respective terms of the projects regulated the work conducted in the Kingdom of Saudi Arabia and Estonia. In general, both projects had to deliver an initial assessment and recommendations for improvement. In the Kingdom of Saudi Arabia, the project concluded with the National Master Plan for Health Data Analytics [3]. In Estonia, the results were integrated into the Person-Centered Integrated Hospital Master Plan [4].

This section describes the framework of definitions and research methods that we developed for the projects.

### Mapping Data Sharing Purposes

Digitalization shall enable gains in the effectiveness of decision-making (better inputs, better decisions) and increase the efficiency of data processing (timely and cost-effective delivery of data), resiliency against missing or erratic data, and sustainability of the data management (agility of the data models and infrastructure). One can assess digital health effectiveness by its ability to generate data (inputs) for decision-making. The World Health Organization (WHO) lists various health care performance indicators for management and policy decisions. The WHO has divided the core health indicators into 4 domains: health status, risk factors, service coverage, and health systems [5]. The system of indicators supports rationalized alignment of priorities and harmonization of investments for various levels of health care systems. We used the system of indicators to model health data completeness. The indicators allowed us to cross-check if the needs of the decision makers were fully met. We analyzed the ability of a health care system to coordinate the data required for the indicators.

The purpose of the collected data is to support health care activities (Figure 1). The activities depend on input data and generate new data, including clinical, managerial, financial, and others. The organizational or human actors of studied ecosystems perform these activities. For example, a hospital manager preparing the financial plans consumes data about the average cost per patient case. On the basis of the WHO indicator domains, the studies searched for evidence of data consumption in public health status and risks, health care activity, resources, funding, and clinical decision-making and research. In the Kingdom of Saudi Arabia case, we paid less attention to the clinical side, mainly focusing on the national public health indicators and the health care system. In the Estonian case, in addition to clinical decision-making, we investigated patient-side decision-making, and patient engagement was considered a separate health care activity.

### Mapping Data Flow

Digital health ecosystems facilitate data flows to resolve data provision and consumption tension. The projects in Kingdom of Saudi Arabia and Estonia mapped the roles, procedures, structures, terminologies, and master data involved in coordinating the flow. We built catalogs of organizations that capture health care data (data providers) and organizations that receive health care data (data consumers). The interoperability between the sources and targets may be organized in many ways, either via bilateral point-to-point agreements or multilateral standards-based agreements. The parties share registries of identifiable objects, such as persons, legal entities, locations, services, and others. We checked the availability of data standards and master data registries. For greater secondary use, multilateral data-sharing agreements shall be in place. If we identified standards-based data sharing, we also examined the governing organization around the standards. Depending on the data governance setup, 1 or more entities would coordinate the data requirements between data consumers and providers, govern information assets, design and enforce data standards, and monitor the continuity of the data flow. The responsibility for the coordination is called data stewardship. The data policy’s task is to regulate the distribution of data governance responsibilities and enable control over them. For example, a data policy may state that licensed health care institutions shall follow the data collection standards set by a single stewardship organization; this may enforce a data flow that makes a log of activities and resources spent on those activities available to health care management and funding stakeholders.

### Mapping Data Sharing

Data sharing is based on the organizational and technical capability that transports data between decision-making locations. We gathered information about the data management platforms, the organizations running the platforms, and the standards supporting data exchange. A data manager or custodian is responsible for maintaining a technical environment and a database structure for data sharing. Regardless of the topology of a data sharing system, centralized or distributed, the data shall be delivered to the correct location at the right time for decision-making. For example, a public registry of laboratory results may transport data between the laboratories, health care providers, and researchers.

The discipline of enterprise information management defines the elements of data flow and sharing. Data governance roles, namely, data policy, data stewardship, and data custodianship, have been used for structuring enterprise information management [6,7]. In the case of the studied projects, we looked for the data governance structures in the national-level health care data organizations.

### Assessing the Maturity of Capability

Various capability maturity models support assessing health care information systems [8]. Many maturity models focus on a specialty; a type of organization; or an area of function (hospital management, diagnostic images, and more). Some models focus on the digital health system’s ability to connect sources and targets of data sharing. The Health Information Sharing Maturity Model (HISMM) from the Mitre Corporation suggests assessing a digital health system from the perspective of 11 capability areas, which cover technology, use, and governance perspectives [9].

The HISMM model has 2 dimensions: 11 characteristics and 5 maturity levels. The maturity level reflects the level of development or goal achievement regarding a capability. If each launch of a data flow requires the creation of a new organization, the flow has a project-based (1) capability level. At the expert-based (2) capability level, existing organizations (experts) can process a data flow. At the standard-based (3) capability level, data flow can be initiated by involving several organizations providing the same level of service. The data flow at the performance-based (4) level constantly produces indicators of the success of its activities. The data flow is at the learning-based or optimizing (5) level if the performance indicators trigger continuous improvement [9]. For the assessment, we enhanced the HISMM levels with the maturity criteria from ISO 33020 and Capability Maturity Model Integration (CMMI), which guide the assessment of capability maturity of processes related to information systems [10,11]. For example, when level 3 of ISO 33020 requires that “a standard process, including appropriate tailoring guidelines, is established and maintained,” we looked for such evidence in our desktop research and in the interview notes.

The characteristics dimension of the HISMM model contains 11 characteristics. The 11 characteristics form a checklist for developing data flows. According to these 11 characteristics, analyzing which additions must be added to the use, technology, or governance organization to increase capacity is possible.

On the basis of our experience with the Kingdom of Saudi Arabia, we introduced the HISMM as an additional tool for assessment in Estonia. Despite our interest, it was economically unreachable for us to redo the assessment in the Kingdom of Saudi Arabia only to compare the HISMM assessment results.

In Estonia, we structured maturity evidence based on the stakeholders’ purpose. The structuring allowed the study to analyze the variation in the inputs collected from different decision makers. For example, we were looking for the differences in the maturity of health care management, clinical decision-making, and patient engagement, where all stakeholders may need data about health care resources.

### The Framework of the Assessments

The complete framework provides categories for mapping the capabilities and assessing the maturity of those capabilities. Textbox 1 below provides a summary of the categories.

The framework drove the capture and analysis of the findings in both projects. We asked the interviewees about the purposes of using health data and the means they used to manage the data. Together with the interview participants, we investigated the stakeholders, information systems, standards, technologies, and platforms on which their data flows were based. For example, we asked the hospitals’ management about the indicators they used in decision-making. Then, we asked the statisticians and IT specialists to describe the sources of the data and the data processing activities.

**The assessment framework includes categories for mapping capabilities from the data and stakeholder purpose perspectives. The purpose is satisfied via data flow and sharing capabilities, which indicate details of the implementation’s maturity level.**

**Data purpose**

- Public health status
- Public health risks
- Health care activity
- Health care resources
- Health care funding
- Clinical decision-making
- Clinical research

**Data flow**

- Data providers
- Data consumers
- Data stewardship
- Data standards
- Data policy
- Master data

**Data sharing**

- Data platforms
- Data custodianship
- Data exchange standards

**Stakeholder purpose**

- Patient engagement
- Clinical decisions
- Health care management
- Research and monitoring
- Health care policy
- Health care funding

**Capability maturity**

- Level 5. Optimizing
- Level 4. Performance
- Level 3. Standards
- Level 2. Experts
- Level 1. Projects

**Capability characteristic**

- Technology

1. Data quality

2. Data transport

3. Data security

4. Interoperability

- Use

5. Usability

6. Alignment

7. Participation

8. Consent

- Governance

9. Data governance

10. Stakeholder governance

11. Sustainability

### Project Activity in the Kingdom of Saudi Arabia

For the situational analysis in the Kingdom of Saudi Arabia, we completed a comprehensive institutional review of the current data systems in the health sector, with an emphasis on how these data were collected and could be routinely made available to the responsible authorities.

We assessed the processes at the institutional, operational, and technical levels. To understand the architectural options for integrated data management, we analyzed the current development plans and statuses, including a rapid assessment of existing computerized information systems, services, and tools. Specifically, we assessed the critical processes for data management and use, system architectures, database architectures, key relevant data sets, data exchange capabilities, geospatial tools, and system platforms available in the health system. The assessment identified the health system’s critical information systems, data sets, and exchange capabilities.

The assessment methodology included primary and secondary sources, including interviews with stakeholders. The research team interviewed policy makers and stakeholders during a sequence of missions in 2019. The stakeholders included the national-level health care coordination (SHC) and management of the sectors, namely, MoH Medical Services, National Guard Medical Services, Ministry of Interior Medical Services, and King Faisal Specialist Hospital & Research Center. The project included an in-depth web search of written information and web-based resources on digital health tools and systems for data exchange and analytics. To improve the primary stakeholders’ capacity and achieve a common understanding of master plan goals, a seminar about global experiences and examples of technical solutions took place during the first technical mission.

### Project Activity in Estonia

The team evaluated Estonian hospitals’ health data and information exchange levels. The method combined interviews and desk research. The aim was to understand the value of health and medical information sharing capabilities to stakeholders, identify gaps in funding, and relate governmental activities to strategies and frameworks.

This rapid assessment used semistructured interviews. Institutional, operational, and technical experts described their view on Estonian digital health care data governance; health data flows; information security; and existing computerized information systems, services, and tools. The analysis considered the hospitals part of a more comprehensive data sharing network. Hence, participants provided inputs about both internal and external data sharing. Specifically, interviews with the stakeholders touched on the critical processes for data management and use, system architectures, database architectures, key relevant data sets, data exchange capabilities, and system platforms available in the health system, considering the current use of the EHIF, the Estonian Health Information System (EHIS), and the NIHD databases.

The data sharing network under discussion included health care institutions, public authorities, and other data users, for example, researchers and patients (from the point of view of hospitals). As the interviews covered the involved participants in both data provider and data consumer roles, the captured evidence also touches on the existing and potential use of data for primary and secondary purposes. On multiple occasions, the interviewed stakeholders were able to share insights into the integration of health data exchange and services with social and labor market services. The assessment covered vital information systems, data sets, and data exchange capabilities of the Estonian health care system.

Altogether 9 stakeholders were interviewed, including hospitals (North Estonia Medical Centre, Tartu University Clinic, Pärnu Hospital, Viljandi Hospital, Põlva Hospital, and East Viru Central Hospital) and specialists from the NIHD, the EHIF, and the Estonian Society of Cardiology. Before the interview, we provided the interviewees with a comprehensive set of questions divided into 11 categories according to the capability attributes of the HISMM. The length of the interviews ranged from 1.5 to 2 hours. Usually, the group consisted of 4 to 5 persons, including the head or vice-head of the institution; chief specialists of clinical, IT, service development departments; health accounting; and statistics.

### Ethical Considerations

This study compiled the framework, methods, and findings from the past project deliverables, which were available publicly or per request from the respective owners. The projects in the Kingdom of Saudi Arabia and Estonia assessed material available from public sources and interviews. The included organizations-appointed interview participants. The projects did not compensate for the participation. We informed the participants about the purpose of the assessment and used the interview results anonymously. The study team never recorded any health data during the interviews or site visits. This study includes statements on the possible limitations of the conclusions.

### Summary of the Methodology

Table 1 summarizes the methods used by the projects in the Kingdom of Saudi Arabia and Estonia.

**Table 1:** Methodology of the case projects.

Methodology element	The Kingdom of Saudi Arabia	Estonia
Research type	Evaluation research	Evaluation research
Research design	Exploratory case study	Exploratory case study
Sampling method	Purposive sampling	Purposive sampling
Data collection method 1	Personal semistructured interviews	Personal semistructured interviews
Data collection method 2	Documentation review	Documentation review
Data analysis method	Qualitative content analysis	Qualitative content analysis
Data coding 1	Data purpose, flow, and sharing	Data purpose, flow, and sharing
Data coding 2	—^a^	Health Information Sharing Maturity Model
Target application	National Master Plan for Health Data Analytics	Person-Centered Integrated Hospital Master Plan, and Information-Sharing Capability Maturity Assessment
Research question	What are the gaps and critical elements for the national-level improvement of the secondary use of health care data?	What are the gaps and critical elements for the national-level improvement of the secondary use of health care data?

^a^Not applicable.

## Results

### Digital Health Landscape in the Kingdom of Saudi Arabia

The Kingdom of Saudi Arabia health care information system encompasses several stakeholders, including primary health care (PHC), hospitals under different jurisdictions, the SHC, the MoH, public health research, quality management, and others. The project looked at the health care system as a whole.

The Kingdom of Saudi Arabia has a population of 35 million, divided between 21.4 million Saudis and 13.6 million non-Saudis. The annual population growth rate was 2.38 in 2020, which dropped slightly from 3.19 in 2010. Part of it can be accounted for by the lowered fertility rate of 1.9 in 2018 and 2.98 in 2010 [12]. The population aged >65 years was 3.4% in 2019, and it is expected to grow to 6% by 2030, which makes it a country with a relatively young population compared with its neighbors in West Asia [13].

The health care system in the Kingdom of Saudi Arabia is mainly funded via the MoH of the Kingdom of Saudi Arabia, which covers 287 hospitals with 45,180 beds, 2257 PHC centers, and 973 specialized medical facilities. In addition to MoH, the governmental health care sector includes providers under the Armed Forces Medical Services, National Guard Medical Services, Ministry of Interior Medical Services, King Faisal Specialist Hospital & Research Center, Royal Commission Hospitals, ARAMCO Hospitals, and Ministry of Education. The total number of hospital beds in other government sectors is 13,989, divided among 50 hospitals. The private sector in the Kingdom of Saudi Arabia runs 167 hospitals with 19,427 beds [14].

In 2000, the Kingdom of Saudi Arabia institutionalized the development of electronic health care as a governmental committee. In 2005, the government established the Saudi Association for Health Informatics, which focused on the growing awareness of electronic health among health care professionals [15]. The effort put into awareness and education has supported the adoption of health IT. Still, the adoption could have been more cohesive, and the use of electronic health systems has been limited [16]. A multiple-case study based on a survey (conducted in 2010) of 6 of the seemingly most advanced medical cities of the Kingdom of Saudi Arabia concluded that inadequate data management policies and procedures, resistance to change, the low analysis of data, and lack of accreditation impact the health IT adoption. The study revealed a need to introduce a national regulator and establish a data exchange plan through a national health information network [17]. The MoH of the Kingdom of Saudi Arabia has invested in the growth of health information exchange on all health care levels. Some researchers have found that the MoH’s focus on information exchange between the health care system participants supports the greater adoption of electronic health records. The sharing adds more value to the data and increases the motivation for quality data capture and improvement of health IT tooling [18].

The SHC, established in 2014, is a successor to the Health Services Council, established in 2002. The role of the SHC is to coordinate and integrate health care stakeholders regardless of the type of ownership or the sector of governance. At the time of the project, the SHC included 16 representatives from several ministries, national health care agencies, education institutions, and health care institutions. The SHC governs some national health centers, including the National Center for Health Information [19].

The National Health Information Center (NHIC) was established in 2013, with the mission to organize health information exchange among all health sectors and related parties, to develop and customize terminology and data exchange standards, to create and supervise telehealth networks, to create national disease registries, and to provide health information to the beneficiaries [20].

### Digital Health Landscape in Estonia

Estonia has a population of 1.3 million, which has declined since 1990. The annual population growth rate has been approaching 0 (from the negative side) in the past years. However, the growth rate has been lifted by migration as the fertility rate of 1.6 per woman is less than that required for reproduction [21]. The population aged >65 years was 20% in 2019 and is expected to grow to 23.5% by 2030, slightly above the average of 18.8% and 21.8% in the region of Northern Europe [22].

All health care institutions operate under private law in Estonia. General practitioners are private entrepreneurs or limited companies. At the same time, hospitals are joint-stock companies or not-for-profit foundations licensed by the Health Board and provide various inpatient or outpatient medical or nursing care. In total, 1428 health care institutions were covered by the National Institute of Health Development statistics in 2019. There are 52 hospitals with 6788 beds, 436 family health centers, 490 dental care providers, 317 specialized outpatient medical care, and others [23]. The health care system in Estonia is governed by the MoSA. The system’s structure includes agencies of the MoSA (eg, State Agency of Medicines, Health Board, NIHD, and Center of Health and Welfare Information Systems); independent public bodies (EHIF); (mainly publicly owned) hospitals under private regulation; private PHC units; and various nongovernmental organizations and professional associations. The financing is organized chiefly through an independent single public-payer EHIF, including ambulance services [24]. The government regulation establishes a list of regional, central, general, local, and rehabilitation hospitals, a total of 19 hospitals, to ensure uniform access to health care services. These hospitals are entitled to receive the necessary construction, renovation, and reprofiling investments from the government budget. With the hospitals mentioned in the list, EHIF concludes treatment financing contracts for at least 5 years based on the type of hospital listed and the corresponding operating license.

The MoSA of Estonia covers public health from the state budget. Private, primarily out-of-the-pocket spending was 22.7% in 2016 [21].

Health care data are divided between 14 primary national-level sources, in-house sources of health care service providers, and databases of research institutions. In addition to inherent health care data sources, the e-government platform enables the secondary use of public registers for health care needs. For example, the Population Register, managed by the Estonian Police and Border Guard Board, is the source of personal data for patient management. The Health Board manages public registers of health care professionals and health care institutions. The State Agency of Medicines manages registers of drugs, medicinal products, and pharmacies. The EHIF collects reimbursement-related health data, registers the status of insured persons, and manages digital prescriptions. The Center of Health and Welfare Information Systems maintains a significant platform for health data sharing, the EHIS [25] that encompasses the whole country, registers all residents’ health history from birth to death, and is based on the e-government infrastructure.

Since 2008, the Digital Health Platform (DHP) has been operating in Estonia, which shares the health care data of the entire country’s residents in a secure e-government environment, both between authorized health care workers and between a health care worker and a natural person. The DHP, whose official name is the EHIS, aims, among other things, to process the data related to the area of health care for entry into and performance of contracts for the provision of health services; for ensuring the quality of health services and the rights of patients; and for the protection of public health, including for the upkeep of registers and the organization of health statistics and the management of health care [24].

### Assessment Results in the Kingdom of Saudi Arabia

In the case of the Kingdom of Saudi Arabia, the project delivered 2 consecutive components that led to the recommendations for decision makers to boost the regulatory, institutional, and technical infrastructure within the country’s health care system, thereby allowing for the efficient collection of health care data.

First, we performed the situational analysis of health and health care data management. The delivered assessment report provided a brief institutional review of the Kingdom of Saudi Arabia health care system’s data management and identified vital information systems, analytical data sets, and data exchange capabilities.

The assessment report revealed that data reporting and analytics procedures, standards, and forms in health care should be coordinated and coherent across the ministries with health care institutions under their jurisdiction and with the MoH and SHC.

On the basis of the review, the report gave recommendations for the long-term institutional, organizational, architectural, and technical redesign of health care data analytics to move from static, fragmented, and incomplete data sets to rapid, reliable, and dynamic data processing, exchange, extraction, and consolidation. We used these recommendations as the basis for the development of the second deliverable, the Master Plan for National Health Data Analytics; it is a policy document that describes the regulatory, institutional, and technical infrastructure that allows the efficient collection of digital health and health care data from health care systems to process and provide appropriate data analytics and business intelligence for policy and decision makers. The SHC of the Kingdom of Saudi Arabia approved the Master Plan in 2020.

The Master Plan outlines a framework for data, roles, and processes. The framework considers analytical data sets, health care indicators, reports, metadata, and catalogs as parts of the data dimension. The framework of roles supports the governance of the data and information flows and endorses the strategic value of the analytical data. The Master Plan defines the specific organizations that shall fulfill the defined roles. The process dimension outlined a set of workstreams for analytical data. It described a path to produce needed policies, objectives, data definitions, analytical product definitions, and standards.

The Master Plan evaluated multiple options for assigning the data governance roles. The final recommendation was to share the responsibilities between the units of the SHC: the policy and strategy management to the SHC Board, data stewardship and analytics to the National Health Analytics Department, data custodianship to the National Health Observatory at the NHIC, and standardization to the Data Standardization Unit at the NHIC.

To operationalize the framework, the Master Plan proposes 3 years to transition responsibilities and develop institutional capacity on all levels. After that, all actors at the national and subnational levels shall adapt to the general and national data analytics frameworks.

### Assessment Results in Estonia

**Figure 2:**
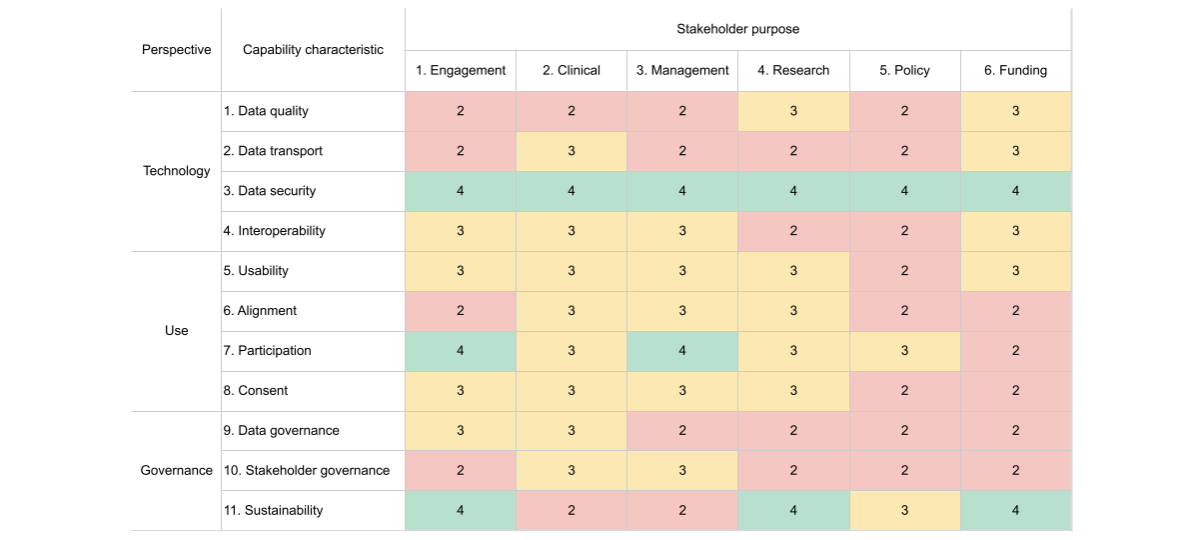
The figure summarizes the Health Information Sharing Maturity Model assessment findings from Estonia. The matrix’s cells depict the maturity levels grouped by the 11 capability characteristics in the vertical dimension and by the 6 stakeholder purposes in the horizontal dimension. The number in the cell shows the corresponding level of maturity, colored red for level 2, yellow for level 3, and green for level 4.

In Estonia, the study used interviews to collect inputs for the assessment. The researchers adopted the HISMM and reorganized the notes using capability attributes and stakeholder purpose. The purpose dimension aimed to summarize the capabilities or flows that the interviews covered. The interview content covered the purpose, flow, and sharing. We asked the interviewees to cover the topics for all the data governance roles of their institutions. For example, depending on a specific capability discussed, a hospital can be a data provider, consumer, custodian, and steward. The notes were analyzed for evidence of maturity and labeled accordingly. The method resulted in a 3D matrix with dimensions for stakeholder purpose, capability attributes, and maturity level (Figure 2).

The researchers estimated the maturity of the information sharing to be on levels 2 to 4. Level 2 represents a situation where the information flow stands on the existing expertise, and the flow outcomes are repeatable. Level 3 indicates the existence of standards and procedures that allow new providers to enter the market. On level 4, the assessed ecosystem shall demonstrate an ability to measure the achievement of the information sharing goals.

**Figure 3:**
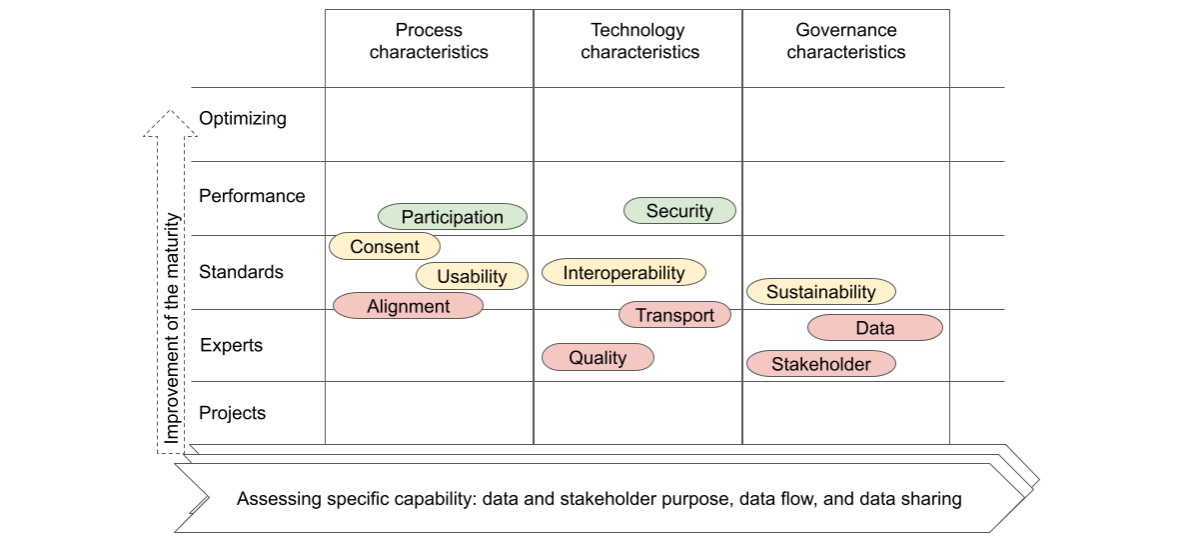
The figure visualizes the maturity levels of Health Information Sharing Maturity Model characteristics in aggregation. It shows that data quality and transport, data and stakeholder governance, and process alignment maturity are lower than other characteristics.

The assessment suggests, also visualized in Figure 3, that the improvement focus should be on data quality, transport, and stakeholder governance. From the perspective of stakeholder purpose, the flows that feed decisions on health care policy show the lowest maturity. The maturity matrix indicates that the data and information may circulate in silos of governance; experts are needed (level 2) to support the data to reach the policy makers. The below-average estimates on data and stakeholder governance hint that the coordination of the data flows is mostly implicit. The interviewees missed the explicit rules and coordination of the secondary use of data. The above-average estimates on data for clinical purposes indicate the success of standardizing patient data flows via the EHIS.

The project in Estonia combined the HISMM into the framework of analytics. The role of the HISMM was to provide insight into the improvement potential of the established flow of data. The findings of the maturity assessment allowed the stakeholders’ viewpoints to be drawn to and the specific characteristics of the flows to be analyzed. The HISMM is a valuable tool for cases where the primary data policy, governance structure, and platform are already in place. Analysis of the specific characteristics provides a basis for targeted improvements. When a data flow misses expectations, an assessment may reveal a specific characteristic that limits the flow. The summary of the HISMM results in Estonia shows the need to improve the focus on data quality, data transport, data and stakeholder governance, and process alignment (Figure 3).

The study in Estonia concluded with a recommendation to align the roles of data providers, consumers, stewards, and custodians for the expanded multipurpose data flows. The current document-based health information sharing model shall transform into a shared space of Integrated Care Records. We reported the assessment results as part of the Person-Centered Integrated Hospital Master Plan, which also included reports of teams of other specialists.

### Comparison of the Assessments

We conducted the assessments according to the framework discussed earlier in this study. To map the elements of data sharing, we interviewed stakeholders using the shared data for the purposes defined by the scope of the projects. We also captured the evidence of the stakeholders interacting with specific data sharing platforms.

The data collection in the Kingdom of Saudi Arabia took place 1 year before the project in Estonia. We mostly replicated the methodological experience from the Kingdom of Saudi Arabia in Estonia, except that we introduced an additional assessment tool, the HISMM (Table 2).

The analysis of the projects in the Kingdom of Saudi Arabia and Estonia demonstrates the challenges of coordinating data flows on a distributed data sharing system. Even if the health care systems in the 2 countries are coordinated differently, in both cases, the conclusions focus on the need to strengthen data flow stewardship (Table 3).

**Figure 4:**
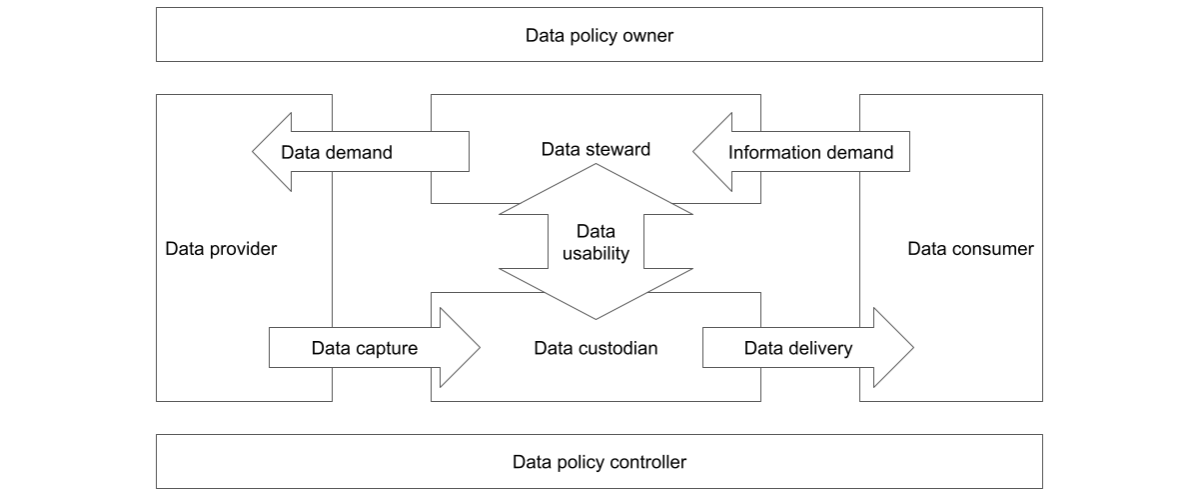
A conceptual overview of the data governance roles and relationships used as a base for mapping the actual organizational structure of the countries. A policy for multiple data use supports resolving demand and supply between data providers and consumers. The policy establishes authority and responsibility for pervasive data stewardship and custodianship.

The conclusions advised the governments to introduce governance policies, which would clarify the responsibilities of the stakeholders. Proper management of the responsibilities of data stewards would increase the value of data providers’ contributions and the value of the custodians’ data. Figure 4 illustrates our conceptual understanding of the data governance roles and their relationships, which we used as a tool to map the roles of the existing or future organizations in our recommendation.

Current data governance in the studied countries follows the vertical model of stewardship, where the data consumers coordinate the information flows for their own needs. Most consumers have also established their own data management or custodian organizations. Notable exceptions are the national databases and message exchange platforms, which support data consumption by multiple institutions. For example, the EHIS manages data consumed by the network of health care providers, patients, and health care registries. A pervasive stewardship function shall increase the secondary use of data.

**Table 2:** The assessments share comparable attributes of scope. The only exception is that the project in Estonia conducted a maturity assessment, which was not part of the project’s scope in the Kingdom of Saudi Arabia.

Attributes of scope	The Kingdom of Saudi Arabia	Estonia
Assessed stakeholder purposes	Clinical, population health, and health care management	Clinical, population health, patient engagement, health care funding, health care management, and health care policy
Assessed data sharing platforms	Hospital EMRs^a^ and analytical data sets	Hospital EMRs, the national EHR^b^, disease registries, and insurance claims registry
Assessed capability maturity characteristics	—^c^	HISMM^d^ technology, use, and governance
Stakeholders interviewed	National-level health care coordination (Saudi Health Council) Management of the sectors (MoH^e^ Medical Services, National Guard Medical Services, Ministry of Interior Medical Services, and King Faisal Specialist Hospital & Research Center)	National-level health care management and policy (Ministry of Social Affairs, North Estonia Medical Centre, Tartu University Clinic, Pärnu Hospital, Viljandi Hospital, Põlva Hospital, East Viru Central Hospital, the National Institute of Health Development, the Estonian Health Insurance Fund, and the Estonian Society of Cardiology)

^a^EMR: electronic medical record.

^b^EHR: electronic health record.

^c^Not applicable.

^d^HISMM: Health Information Sharing Maturity Model.

^e^MoH: Ministry of Health.

**Table 3:** The findings from the 2 countries demonstrate similarities in the expected achievements, identified barriers, opportunities, and principal conclusions. Regardless of the digitization of workplaces in both cases and sophisticated data integration solutions in the Estonian case, siloed data stewardship limits the multiuse of health data.

Attributes of findings	The Kingdom of Saudi Arabia	Estonia
Expected achievement	Timely and efficient delivery of health care system and public health indicators, and standard and special reports	Timely and efficient decision support for clinical, management, and financial decisions
Barriers	Lack of interoperability standards and siloed sectoral stewardship	Siloed vertical stewardship
Opportunities	Digitized workplaces in health care and cross-sectoral health care governance structures (SHC^a^)	Digitized workplaces in health care, secure integration platform (XRoad), national EHR^b^ (EHIS^c^), data and data exchange standards, and national-level clinical decision support
Principal conclusions	Align the roles of the stakeholders and engage the participants in a standardized data flow	Align the roles of stakeholders and standardize the event-driven sharing of EMRs^d^

^a^SHC: Saudi Health Council.

^b^EHR: electronic health record.

^c^EHIS: Estonian Health Information System.

^d^EMR: electronic medical record.

## Discussion

### Principal Findings

The study maps the common thread of thought from 2 distinct projects in the Kingdom of Saudi Arabia and Estonia, assessing the potential of secondary use of health care data and supporting governmental decisions. The projects apply comparable frameworks and methods, allowing the comparison of the barriers, opportunities, and conclusions. The findings include both the frameworks used and the conclusions made. The framework of assessment defines 2 dimensions of analysis. First, there is the need to identify and improve data sharing flows between data providers and consumers. The analysis investigates multiple purposes of data, data and exchange standards, stakeholders’ governance, and shared data management platforms. Second, the framework considers the maturity of the data sharing implementation. The maturity assessment measures the level of institutionalization of health data sharing. The second part of the framework was included and applied only to the project in Estonia.

The assessment revealed opportunities and barriers in the secondary use of health data. Starting with the opportunities, the included institutions demonstrated high levels of digitization in the workplace. In the Estonian case, we also experienced advanced integration platforms and interoperability standards implemented nationally. The latter has supported the development of sophisticated solutions for national electronic health record and clinical decision support. However, the countries have maintained a fragmented organization of data stewardship, which has not been able to coordinate the need for data. In both cases, the assessments concluded with a recommendation to implement pervasive data stewardship to align the need for data.

While many countries have digitalized information necessary for clinical work and described the data relatively well, especially in most European countries and North America, unified routines and applications for the secondary use of digital data in health care are largely still being planned.

In Sweden, more than 100 health care quality registries collect individual-based clinical data for research and improvement of health care delivery [26]. In Estonia, 6 medical registries and databases collect, process, and distribute data about health and medicine [27]. Studies propose that clinical quality registers can be cost-effective and yield significant investment returns [28]. The number and quality of the registries indicate success in the secondary use of health care data. The registries also introduce data capture, integration, and delivery costs for secondary use. These professional specialty or national quality registries are often developed and managed in silos, leading to high maintenance costs and challenges in interoperability.

Regardless of the advanced information systems in hospitals, the health care system in the Kingdom of Saudi Arabia spends considerable time and resources collecting statistical data. There is much manual processing due to the lack of standards for integration and semantics. The same applies to Estonia; despite the common health data interoperability standards and transport system, secondary use of health care data is often in silos and needs additional effort. In the Kingdom of Saudi Arabia and Estonia, the data consumers coordinate their needs directly with the data providers, reinforcing the traditional model of form-based reporting. The form-based approach introduces duplication at the data capture; one may call it secondary capture. To avoid resource wasting and duplication, collecting the data consumers’ need shall be part of the standards of primary data capture.

In Estonia, the advanced semantic interoperability of the clinical documents shared via the EHIS enables the automation of clinical decision support [29-32]. Such features include drug-drug interaction alerting, context-driven suggestions of clinical guidelines, and automatic patient summaries based on clinical documents. These features increase the use of data but only inside the vertical of clinical decision-making. The EHIS could also facilitate data flows for public health, health care management, and clinical research decision-making.

The stakeholders of health care data need to cooperate through a strategic digitalization process. Often, the participants are not aware of the discontinuity of the data flow. The study in Estonia indicated that the participants were relatively satisfied with the data management tools and their engagement in the flow. Instead, they reported problems with data quality and governance. The users expressed their frustration regarding duplicate data capture but could not relate it to the low alignment of the processes. We hypothesize that the interview results indicate disruptions in the data flow. The respondents struggled to find source data to fill in the data entry forms for secondary use. Designing and managing a flow that connects data capture with a single consumer is relatively easy, ensuring satisfaction with the tools and participation. Only a helicopter view of the landscape of data needs shows the shortcomings of governance and the chronic waste of capturing the same data repeatedly. Efficiency in the secondary use of data starts from the health care policy establishing clear goals and management.

Single-purpose capture of data and single-purpose databases are indicators of the low secondary use of health care data. The data flow design should follow the principle of “collect once and use multiple times.” The studied cases reveal the barriers built between the domains of information purpose. The health care system extends over 6 ministries in the Kingdom of Saudi Arabia. It takes a long time and heavy work to combine data across the borders of the governance verticals. In Estonia, where the organization is more straightforward, data collection for different purposes is still split between different data consumers, resulting in independent data flow designs without proper interoperability. For example, health care providers must simultaneously record the exact data for clinical decisions, management, funding, statistics, and research. The new policy shall require the unification of the demand of data consumers into a single standard of data capture.

The analysis of health data sharing challenges in the Kingdom of Saudi Arabia and Estonia demonstrates that the digitization of the workplaces, integration of information systems, and advanced semantic interoperability are insufficient for secondary use on a large scale. A prerequisite for secondary use is a health care policy that emphasizes the need for the continuity of the data flow. The health policy should address governance of the data and stakeholders without introducing central bottlenecks for innovation. The policy should guide parties to map the impacts of their data processing and increase the value of their data through greater secondary use. A health data sharing system shall reward the measurable secondary use of data assets.

Advances in the digitization of health data and integration of information systems open the way to the digitalization of health care processes. Shared data enables the coordination of activities of a digital process. Stewards and custodians must govern health care data through the diverse organizations, workplaces, and information systems landscape. Data governance conceptualizes and carries out stewardship responsibilities based on data access, custodianship, and use policies [33]. The conceptual framework for data governance by Abraham et al [6] suggests structural, procedural, and relational mechanisms. The structural and relational mechanisms include establishing clear organizational responsibility and communication. The governments in demand for greater secondary use of health care data shall establish data policy with precise coordinating mechanisms.

We saw that digitalizing data providers and consumers is insufficient for efficient secondary use of data. There is a need for a DHP that enables data and information sharing. However, having a DHP only for clinical data is insufficient. For secondary use, the stewardship must include the requirements of all targeted consumers. This recommendation is also very much in line with the observation from a 2021 report that calls for more substantial public-patient participation in the secondary use of health data [2].

There is an ever-growing demand for better data and information for decision-making. Modern health care and research depend on data from various domains, including education, environment, and social care. It is an ongoing effort to analyze and integrate the new demand for data.

### Limitations

The study reports the findings from 2 projects from 2 countries. The findings present certain commonalities but still have a limited generalizability for different contexts. Countries or regions searching for advice may present circumstances that demand noticeably different strategies for their digital health improvement. It is also essential to understand the role of the frameworks when trying to replicate the results. For practical reasons, a solid framework is essential for such projects, as effective planning, execution, and analysis require a rigid structure. However, the choice of a framework indicates the researcher’s focus and may lead to a limited space of findings. The studied projects develop policy suggestions for health data governance on the national level. Controlled empirical validation of the suggested policies is nearly impossible. The conclusions mainly depend on the internal validity of the research, where we build on the experience of the involved stakeholders and findings from similar experiments.

### Conclusions

In this study, we have analyzed 2 projects that assessed and provided advice for the national-level improvement of the secondary use of health care data. The study provided an overview of the projects’ backgrounds, frameworks, methods, and results. Finally, we discussed the main advice from the projects. The study shows that 2 high-income countries with very different health care systems have comparable issues with the secondary use of health care data. National-level secondary use shall build on an overarching data policy that enables horizontal stewardship to coordinate requirements of a diverse landscape of health care data consumers.

## Abbreviations

CMMI: Capability Maturity Model Integration
DHP: Digital Health Platform
EHIF: Estonian Health Insurance Fund
EHIS: Estonian Health Information System
HISMM: Health Information Sharing Maturity Model
MoH: Ministry of Health
MoSA: Ministry of Social Affairs
NHIC: National Health Information Center
NIHD: National Institute for Health Development
PHC: primary health care
SHC: Saudi Health Council
WHO: World Health Organization
